# *Chlamydia trachomatis*: a model for intracellular bacterial parasitism

**DOI:** 10.1128/jb.00361-24

**Published:** 2025-02-20

**Authors:** Erin P. Smith, Raphael H. Valdivia

**Affiliations:** 1 Department of Integrative Immunobiology, Duke University School of Medicine 12277 , Durham, North Carolina, USA; 2 Center for Host-Microbe Interactions, Duke University School of Medicine 12277 , Durham, North Carolina, USA; Geisel School of Medicine at Dartmouth, Hanover, New Hampshire, USA

**Keywords:** *Chlamydia*, obligate intracellular pathogen, host tropism, inclusion membrane proteins (Incs), lateral gene transfer, bacterial persistence, genetic tools, model organism

## Abstract

*Chlamydia* comprises a diverse group of obligate intracellular bacteria that cause infections in animals, including humans. These organisms share fascinating biology, including distinct developmental stages, non-canonical cell surface structures, and adaptations to intracellular parasitism. *Chlamydia trachomatis* is of particular interest due to its significant clinical importance, causing both ocular and sexually transmitted infections. The strain L2/434/Bu, responsible for lymphogranuloma venereum, is the most common strain used to study chlamydial molecular and cell biology because it grows readily in cell culture and is amenable to genetic manipulation. Indeed, this strain has enabled researchers to tackle fundamental questions about the molecular mechanisms underlying *Chlamydia’s* developmental transitions and biphasic lifecycle and cellular adaptations to obligate intracellular parasitism, including characterizing numerous conserved virulence genes and defining immune responses. However, L2/434/Bu is not representative of *C. trachomatis* strains that cause urogenital infections in humans, limiting its utility in addressing questions of host tropism and immune evasion in reproductive organs. Recent research efforts are shifting toward understanding the unique attributes of more clinically relevant *C. trachomatis* genovars.

## INTRODUCTION

*Chlamydia trachomatis* is a bacterial pathogen with a significant impact on human health, as it is the leading cause of bacterial sexually transmitted infections and preventable blindness (1, 2). Because of its obligate intracellular lifestyle and complex developmental cycle, *C. trachomatis* has been a difficult pathogen to study. As a result, research has historically gravitated toward easy-to-grow, genetically tractable strains like *C. trachomatis* L2/434/Bu to understand fundamental aspects of *Chlamydia* molecular and cellular biology that would otherwise be challenging to achieve in strains that are more relevant to human diseases (3, 4). Indeed, with the introduction of genetic tools, *C. trachomatis* strain L2/434/Bu, hereafter referred to as “L2,” has provided critical insights into the unique biphasic lifecycle of *Chlamydia*, elucidating mechanisms of *Chlamydia* inclusion development (5, 6). It also enabled the identification of strategies that allow *Chlamydia* to invade mammalian cells and thrive as an obligate intracellular pathogen, including strategies to evade cell-autonomous defense mechanisms and maintain infection persistence (7). These characteristics have made L2 instrumental in studying both the basic biology and virulence mechanisms of *C. trachomatis*, including the more medically prevalent urogenital and ocular strains. However, the usefulness of *C. trachomatis* L2 is also limited, particularly when modeling urogenital infections.

## HISTORICAL CONTEXT, EPIDEMIOLOGICAL IMPACT, AND TAXONOMIC DIVERSITY OF *C. TRACHOMATIS*

The discovery of *C. trachomatis* in the early 20th century marked the beginning of efforts to understand this unique pathogen (8). Initially misclassified as a virus, *Chlamydia* was recognized as a bacterium only in the mid-1960s (9). Halberstaedter and Prowazek (8) identified the organisms as cytoplasmic inclusion bodies in ocular epithelial scrapings from orangutans inoculated with trachoma patient material, initially naming them “Chlamydozoa.” In the 1930s, microscopic observations led to the first description of *Chlamydia*’s intracellular developmental cycle (10). However, isolating the trachoma agent proved challenging, and it was not until 1955 that Tang and colleagues (11,12) successfully adopted a protocol developed for *Rickettsia* using embryonated chicken eggs to isolate the trachoma agent (12). Tang proposed that the trachoma agent was a virus based on its inability to grow in bacteriological media and its continued infectivity after passage through a 0.4 µm membrane filter. However, he noted several key features: the trachoma “virus” was unusually large, had structures resembling bacterial capsules, and was susceptible to multiple antibiotics. These observations laid the groundwork for modern *Chlamydia* research.

Due to its small size and inability to be cultured outside of cells, the classification of *Chlamydia* was debated for many years, with researchers proposing it to be a protozoan, virus, viral-bacterial hybrid, or mycobacterium-like. The prevailing consensus for some time considered this microbe as a “large virus,” which led to the use of terminology from virology, such as “inclusions,” to describe *Chlamydia* structures (9). It was not until 1966 that *Chlamydia* was definitively established as a bacterium. Today, it is well recognized that bacteria of the genus *Chlamydia* are obligate intracellular pathogens characterized by distinctive developmental stages alternating between replicative and infectious forms. Genomic analysis estimates that members of this genus co-evolved with eukaryotic cells for over half a billion years when metazoans first emerged (13–17). This long-term adaptation to intracellular growth niches has led to significantly reduced *Chlamydia* genomes, with a relative lack of pseudogenes and a marked dependence on the host for essential nutrients.

*C. trachomatis* was first associated with sexually transmitted infection (STI) in the 1970s. This recognition significantly impacted public health approaches, leading to increased awareness, screening, early detection programs, and treatment efforts. Since then, it has become the most frequently reported bacterial STI globally (1). The United States ranks among the developed countries with the highest STI rates, with newly diagnosed *C. trachomatis* cases exceeding 1.6 million in 2021 (18, 19). Various factors, including reduced condom usage, diminished screening and treatment efforts, and limited healthcare access for underserved communities, drive these rates (1). Given the asymptomatic nature of many infections, *C. trachomatis* infections are likely significantly higher than reported (20). The effects of infection are particularly detrimental to women; untreated *Chlamydia* infections can lead to chronic conditions, such as pelvic inflammatory disease and salpingitis, and ultimately, ectopic pregnancies and infertility (21). Early diagnosis and treatment are crucial to prevent these severe outcomes, underscoring the importance of public health interventions, especially awareness campaigns targeting at-risk populations.

*C. trachomatis* is also the etiological agent of trachoma, a communicable eye disease and the leading cause of preventable infectious blindness. The term trachoma, meaning “roughness” in Greek, describes the appearance of the conjunctiva following infection (2). During the Napoleonic era, trachoma likely spread to Europe as troops returned from North Africa and remained endemic in parts of Europe until the beginning of the 20th century (22). Today, trachoma is endemic in densely populated regions of sub-Saharan Africa and Asia due to limited healthcare and inadequate sanitation. Approximately two million people globally are visually impaired by trachoma infections (2, 23).

A connection between ocular and venereal chlamydial diseases was first recognized in 1911 by Lindner (24), who observed similarities between the cytoplasmic inclusions in ocular infections in newborns and cervical scrapings from their mothers. This finding was significant as it highlighted the ability of the same pathogen to infect different tissue types and provided insights into *Chlamydia* pathogenesis and transmission. Further parallels between venereal diseases like blennorrhoea, lymphogranuloma venereum (LGV), nongonococcal urethritis, and trachoma conjunctivitis were proposed in 1939 by Harrison and Worms (25), who suggested that the same infectious agent caused these diseases. While written records of trachoma date back thousands of years (26, 27), the history of *C. trachomatis* as the cause of a STI is less documented and more complex (28).

## PHYLOGENY AND DIVERSITY OF *C. TRACHOMATIS*

The genus *Chlamydia* was proposed in 1966, with taxonomy based on its obligate intracellular lifestyle and biphasic developmental cycle (29). *C. trachomatis* comprises numerous genovars (30), all implicated in human diseases. These genovars are divided into three major pathotypes: the ocular tropic genovars (A–C), associated with trachoma; the genitourinary tract tropic genovars (D–K), which primarily cause sexually transmitted infections; and the more invasive LGV genovars (L1–L3), which can spread to lymph nodes and cause systemic infection.

Cultivating chlamydial organisms has historically proven challenging. One LGV isolate (strain L2) from an inguinal bubo of an infected human male in 1969 (3) proved easier to cultivate, exhibited favorable growth characteristics, and demonstrated notable virulence, including lethality in mice following low titer inoculations via intracerebral or intranasal routes (3, 31). As a result, this strain rapidly became the default choice for many molecular and cell biological applications. Additionally, L2 was one of the first fully sequenced *C. trachomatis* genovars, which, along with its genetic tractability, solidified its use as a model organism for applying biochemical approaches and developing molecular genetic tools for *Chlamydia* (32).

*Chlamydia* genomes are compact, typically around 1 Mb, with 850–1,100 genes (33). This compactness reflects the organism’s significant dependence on host cells for nutrients and metabolic intermediates, as many essential functions have been lost. Apart from a “plasticity zone” (PZ) near the chromosomal replication terminus, the genetic content and gene organization are highly conserved across *Chlamydia*. Most *Chlamydia* species infect mucosal surfaces, but their specific sites of infection and host tropisms vary, influencing disease outcomes and immune responses (34). *C. trachomatis* infects humans exclusively, and differences in tissue tropism among its genovars correlate with variations in polymorphic membrane proteins (Pmps), inclusion membrane proteins (Inc), cytotoxins, and metabolic pathways, such as those for tryptophan and biotin biosynthesis (33, 35–38). Table 1 summarizes key differences between *Chlamydia* species.

**TABLE 1:** Comparison of key characteristics among *Chlamydia* species

Characteristic	*C. trachomatis* ocular genovars^*a*^	*C. trachomatis* urogenital genovars^*b*^	*C. trachomatis* LGV genovars^*c*^	*Chlamydia muridarum*	*Chlamydia psittaci*	*Chlamydia pneumoniae*	References and further readings
Median genome size (Mb)	1.04	1.04	1.04	1.07	11.17	1.23	(33)
Median number of CDS	903	906	897	905	977	1,046	(33)
Plasmid (~7.5 kb)	Present	Present in most strains	Present in most strains	Present	Present in most strains	Present in animal strains and absent in human strains	(39–41)
Plasticity zone components	MAC^*d*^/perforin, PLD^*e*^ (six genes), cytotoxin (fragments, most non-functional), tryptophan operon (partial)	MAC/perforin, PLD (six genes), cytotoxin (fragments, functional), tryptophan operon (trpRBA)	MAC/perforin, PLD (six genes), tryptophan operon (trpRBA)	MAC/perforin, PLD (nine genes), cytotoxin (three genes), purine interconversion	MAC/perforin (partial or absent in some strains), PLD (two genes), cytotoxin (one gene, partial in some strains), purine interconversion (absent in some strains)	MAC/perforin (truncated in human isolates), PLD (two genes), purine interconversion (some strains)	(33, 35, 41–43)
Number of Pmps	9 genes	9 genes	9 genes	9 genes	18–25 genes	21–24 genes	(33, 44)
Inc-like proteins	65–69	65–69	65–69	65–66	102–108	140–147	(33)
Host specificity	Humans	Humans	Humans	Rodents	Primarily birds; zoonotic transmission to humans and other mammals	Humans, mammals, reptiles, and amphibians (strains are host specific)	(45, 46)
Primary tissue specificity	Ocular	Ano-urogenital	Ano-urogenital and systemic	Gastrointestinal and respiratory	Respiratory, gastrointestinal, reproductive, and systemic	Respiratory and vascular	(34, 47–49)
Bacterial egress mechanisms	Extrusion and lysis	Extrusion and lysis	Lysis (high), extrusion, and *Chlamydia*-containing spheres (low)	Extrusion (high) and lysis	Lysis and *Chlamydia*-containing spheres	Extrusion (high) and lysis	(50–54)
Sensitivity to IFNγ	Sensitive to IDO^*f*^, persistence or death in humans; IRG^*g*^ killing in mice	Resistant to IDO, persistence in humans; IRG killing in mice	Resistant to IDO, persistence in humans; IRG killing in mice	Highly sensitive to IDO, death in humans; resistant to IFNγ-mediated clearance in mice	IDO restricts growth in human cells; IRGs restrict growth in mice	IDO induced persistence in human cells	(55–59)
Animal Infection Models	Non-human primates (cynomolgus macaques)	Mice, non-human primates (pig-tailed and rhesus macaques)	Mice, non-human primates (pig-tailed and rhesus macaques)	Mice	Mice, birds (e.g., chickens), bovine	Mice, rabbits, non-human primates (cynomolgus and rhesus macaques)	(60–64)

^*a*^ ocular genovars include A-C.

^*b*^ urogenital genovars include D-K.

^*c*^ LGV genovars include L1-L3.

^*d*^ MAC, membrane attack complex.

^*e*^ PLD, phospholipase D.

^*f*^ IDO, indolamine 2,3-dioxygenase.

^*g*^ IRG, immunity-related GTPases.

Most *Chlamydia* strains also possess a highly conserved plasmid that is important for bacterial virulence (45, 65). The plasmid encodes eight protein-coding genes (*pgp*) and two small antisense RNAs (45, 65–68). In addition to plasmid regulatory genes, several plasmid genes contribute to infectivity. Pgp3 is a virulence-associated effector, while Pgp4 is a transcriptional regulator of chromosomal virulence factors (69). Plasmid-deficient *C. trachomatis* strains, particularly those lacking *pgp3* and *pgp4*, can still infect hosts but result in reduced pathology (70–73).

## DISTINCTIVE CHARACTERISTICS OF *CHLAMYDIA*: A MODEL FOR INTRACELLULAR PATHOGENS

**Fig 1:**
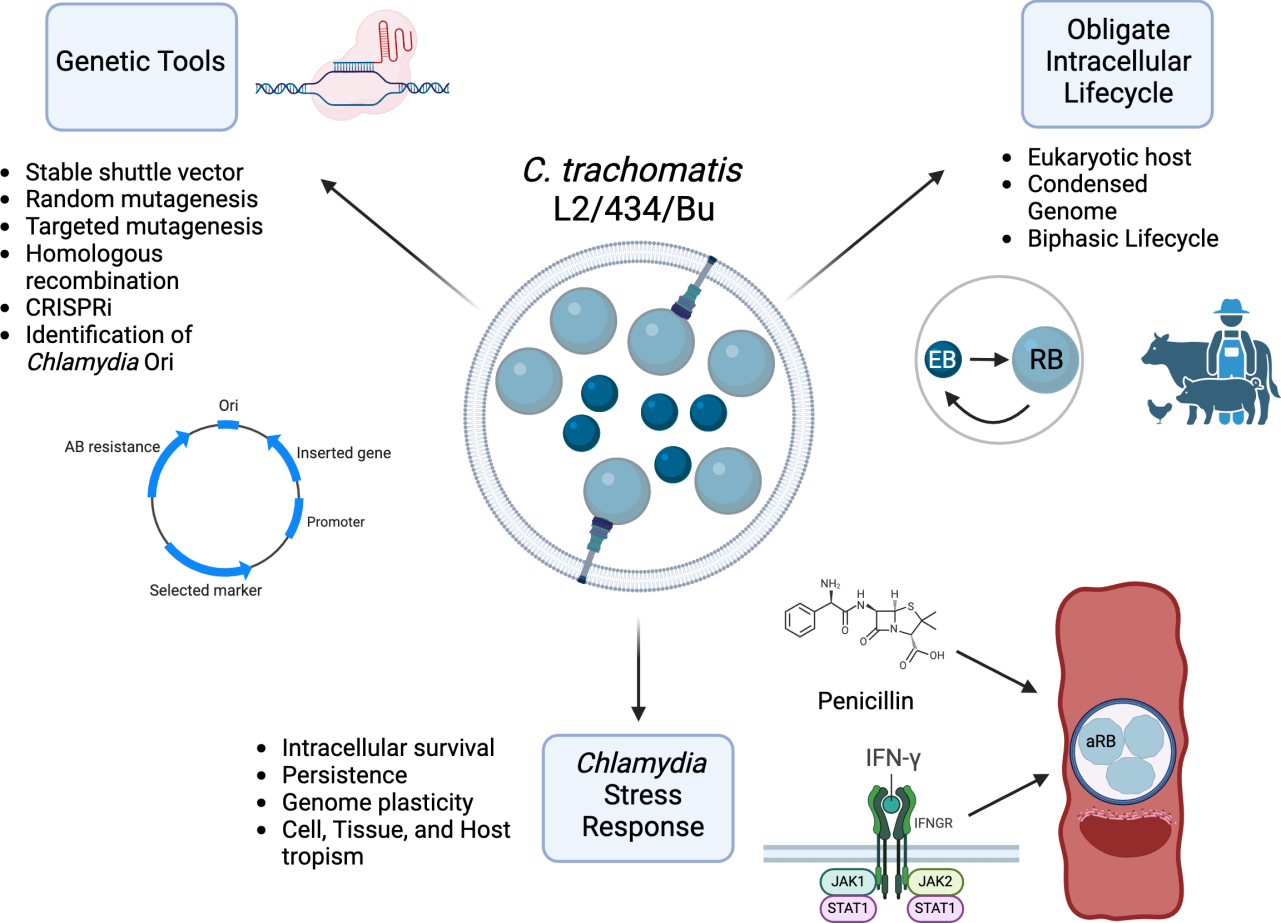
*C. trachomatis* L2/434/Bu as a model organism for chlamydial research. This figure highlights three key aspects of *C. trachomatis* L2/434/Bu that have significantly advanced our understanding of chlamydial biology. The obligate intracellular lifecycle and stress response mechanisms, which are largely conserved throughout the *Chlamydia* genus, have been studied extensively using L2/434/Bu, making it a foundational model for these traits. Furthermore, the development of genetic tools, predominantly achieved in L2, has been instrumental in advancing *Chlamydia* research and can be adapted for studying other species. Created in https://BioRender.com.

Unlike facultative intracellular bacterial pathogens, such as *Salmonella enterica* or *Mycobacterium tuberculosis*, *Chlamydia* requires a host cell for survival and replication under all conditions, making traditional microbiological techniques challenging. Nonetheless, the isolation and subsequent studies of *C. trachomatis* L2, including the generation of genetic tools, have accelerated research on these bacteria. *C. trachomatis* L2 has become a model organism for investigating unique aspects of *Chlamydia* biology, specifically its intracellular lifestyle and stress response (Fig. 1).

### Chlamydial developmental biology

The cataloging of *Chlamydia* developmental forms dates back to observations made in the 1930s in various *Chlamydia* species (10, 74–82). However, *C. trachomatis* L2 has been instrumental in characterizing at the molecular level the unique developmental transitions (5, 6, 83) between the infectious elementary body (EB) and the replicative reticulate body (RB) forms (5, 84).

The *Chlamydia* infectious cycle typically ranges from 48 to 96 hours. It begins with EBs, which are approximately 250 nm in diameter and appear as electron-dense cocci (74–76). EBs have a compact morphology, mediated by chromatin condensation facilitated by histone-like proteins HctA and HctB (85, 86). Unlike most bacteria, EBs contain low levels of peptidoglycan in their cell envelope (87), a critical structural component of bacteria. Instead, EBs compensate by extensively cross-linking cysteine-rich proteins (OmcB and OmcA) with the outer cell membrane (87–90). Notably, EBs are pre-primed with type III secretion system (T3SS) effectors that facilitate invasion and inclusion formation (91–95).

The invasion of host cells by EB forms depends on the rearrangement of actin, a process initialized by the secretion of T3SS effectors like TarP. TarP functions as both an actin nucleator through its WASP-homology 2 (WH2)-like domains and as a signaling amplifier by recruiting host proteins such as Rac1 to facilitate cytoskeletal remodeling (93, 96). Upon invading host cells, EBs reside within endocytic compartments rapidly modified to form pathogen-containing vacuoles, known as “inclusions” (97–99). EBs rapidly differentiate into RBs that express a new set of virulence effectors (100), including a large family of proteins (Inc) that insert into the inclusion membrane to evade degradation of the inclusion and modulate host vesicular transport and cytoskeletal function (101–107). RBs then undergo multiple rounds of replication in the inclusion.

As the cycle progresses, RBs asynchronously differentiate back into EBs (108, 109), a process influenced by peptidoglycan deposition and changes in bacterial cell size (110, 111). Studies have indicated peptidoglycan deposition in live bacteria involves a mixed growth mechanism, including bud formation and localized growth sites, rather than binary division (110, 112, 113). The release of EBs from the host cell occurs either through lysis or non-lytic egress of inclusions, allowing the bacteria to infect neighboring cells (Table 1 [50, 51, 114]. When multiple EBs enter the same host cell, inclusions fuse into a single inclusion, a process dependent on *Chlamydia* Inc proteins (115, 116).

Despite significant advances, the mechanisms controlling fate decisions in *Chlamydia*, particularly the regulation of EB and RB conversion, remain poorly understood. C. *trachomatis* L2 secretes over 30 Inc proteins, and their functions are just beginning to be elucidated through bacterial mutagenesis and transgene expression in mammalian cells (117–121).

### Molecular genetic tools for *C. trachomatis* L2

Stable transformation of *Chlamydia* remained elusive for decades. This challenge was overcome with the generation of the first shuttle plasmid containing the *C. trachomatis* L2 plasmid (pL2) and a ColE1 origin of replication (122), along with the optimization of transformation protocols. The incorporation of a β-lactamase (*bla*) or chloramphenicol acetyltransferase (*cat*) resistance genes in the plasmid allowed for the selection of transformants after pre-incubation of EBs with DNA and CaCl_2_, followed by infection of mammalian cells. Shuttle vectors based on the *C. trachomatis* genovar E plasmid (pSW2), pL2, and their derivatives are the most used expression vectors in *C. trachomatis* research (123, 124). These plasmids have enabled the expression of genes encoding fluorescent reporters, gene overexpression, and genetic complementation of mutants created by other methods (e.g., ethyl methanesulfonate [EMS]) (125). EMS-generated *C. trachomatis* mutant libraries have been instrumental for both forward and reverse genetic approaches, identifying key virulence factors like InaC, a regulator of F-actin assembly and Golgi reorganization around the inclusion; CpoS, a Rab-scaffolding protein that maintains inclusion membrane stability; and GarD, which prevents ubiquitination of inclusions in response to IFNγ (126–128). Chemical mutagenesis has also enabled the identification and isolation of temperature-sensitive alleles of various *C. trachomatis* mutants (129).

The development of chemical transformation methods for *C. trachomatis* facilitated the use of insertional inactivation tools for chromosomal genes. One such tool is mobile group II introns (TargeTron), which direct the integration of reverse-transcribed DNA into specific genome sites (130). This method was initially used to disrupt the gene encoding inclusion membrane protein A (IncA), which is required for homotypic fusion of inclusions (115, 131, 132). Since then, this technique has been applied to inactivate multiple non-essential genes in *C. trachomatis* and other *Chlamydia* species (127, 133–135). Similarly, transposon mutagenesis was achieved using a Himar1 transposon system adapted for *C. trachomatis* L2. However, this random insertional mutagenesis approach remains underutilized due to the low efficiency of chemical transformation, though it has yielded 81 unique transposon mutants and 54 gene disruptions (136).

Subsequent advancements led to the development of plasmid vectors suitable for allelic exchange, including the fluorescence-reported allelic exchange mutagenesis (FRAEM) technique (137). In this method, the *Chlamydia* plasmid maintenance gene *pgp6* is controlled by a tetracycline-inducible promoter, enabling the plasmid to function as a suicide vector. The plasmid, containing β-lactam resistance and GFP genes flanked by the target gene’s sequences, integrates into homologous chromosomal regions, replacing the gene of interest with these reporter elements. An additional fluorescent reporter (mCherry) in the plasmid backbone allows successful gene replacement events to be screened. This tool has been used for targeted gene knockouts of virulence factors, including *tmeA*, *tmeB*, *tarp*, and *trpA* in *C. trachomatis* L2 (138, 139). It was later adapted to include Cre recombinase recognition sites flanking the *gfp-bla* cassette (140), facilitating marker-less mutations and reducing polar effects on downstream genes. Another recent tool adapted for homologous recombination in *Chlamydia* is the λ Red system, which uses three bacteriophage proteins to process plasmid DNA into a linear fragment with single-stranded overhangs for genome integration (141, 142). Like FRAEM, the target gene’s flanking sequences encompass GFP and a chloramphenicol resistance gene. This system enables the deletion of single or multiple neighboring genes, aiding in studying individual genes within operons. Unlike other plasmids, this vector lacks *Chlamydia* maintenance genes, making it suitable for use across *Chlamydia* species (142).

Additional tools include CRISPR interference (CRISPRi) for the inducible repression of essential genes. The first *Chlamydia* CRISPRi system employed the inducible expression of a catalytically inactive Cas9 (dCas9) and a guide RNA (gRNA) that target chromosomal sequences (143). The expression of dCas9 and gRNA targeting the gene *incA* successfully inhibited protein expression, resulting in the expected fragmented inclusion phenotype (144). This system’s stability was enhanced by switching to dCas12 and refined to target multiple genes simultaneously (145).

The maintenance of *Chlamydia* plasmids is species-restricted, primarily due to variations in the *Chlamydia* origin of replication sequences among species. As a result, shuttle vectors developed for *C. trachomatis* cannot be used in other *Chlamydia* species. However, the concepts learned from *C. trachomatis* vector development have facilitated the generation of shuttle vectors for *Chlamydia muridarum*, *Chlamydia psittaci*, and *Chlamydia pneumoniae* using sequences from their endogenous plasmids (146–148). Recently, a 22-bp tandem repeat, identified as the *Chlamydia* plasmid origin of replication, was determined sufficient for species-specific episomal maintenance (149). This significantly expands the genetic toolkit available to all *Chlamydia* spp., especially for clinically relevant strains that remained genetically intractable. Developing and implementing genetic tools for other *C. trachomatis* genovars will be critical to identifying genes responsible for host and tissue tropism and disease severity.

### Mechanisms and implications of lateral gene transfer in *Chlamydia*

Due to their obligate intracellular nature, *Chlamydia* was initially thought to have a limited capacity for genetic exchange. However, it is now apparent that *Chlamydia* may have acquired a significant portion of their genomes—about 8%–30%, depending on the species—through lateral gene transfer (LGT), with a higher incidence among zoonotic species (150). Most LGT within the Chlamydiae phylum appears to occur within species, likely due to the restricted ecological niches. For *C. trachomatis*, intraspecies gene transfer was first reported in the 1990s and later confirmed to be widespread in clinical studies, affecting strains with different tissue tropisms and virulence (151–153). Intraspecies LGT occurs at a much higher frequency than interspecies recombination, leading to the exchange of large DNA segments (154). Although *in vitro* studies suggest that co-infection of a single cell may be required for LGT, cohabitation within a single inclusion appears not essential (155).

Gene transfer can involve significant portions of the *C. trachomatis* chromosome (156). *Chlamydia* plasmids do not encode conjugation machinery, making natural transformation the most plausible route for DNA acquisition, especially since homologs of competence genes are encoded in the genome (136, 157, 158). The spread of antibiotic resistance through LGT is a recognized concern, as notably observed in *C. suis*, which acquired the tetracycline efflux pump gene, *tetA(C*), through a transposon-mediated process (159).

Recombination in the setting of co-infections has been leveraged to perform genetic mapping in *C. trachomatis* and *C. muridarum* (42, 155, 156). Before protocols for targeted mutagenesis in *C. trachomatis* were developed, associations between chemically derived mutations and mutant phenotypes could be mapped by homologous recombination following LGT in *C. trachomatis* L2 (160). Natural homologous recombination has been used to identify hotspots for LGT, both intraspecies between *C. trachomatis* genovars and interspecies between *C. trachomatis* and *C. muridarum*. Cells co-infected with *C. muridarum* mapping strains, created through the random insertion of a chloramphenicol-resistance cassette by transposon mutagenesis, and *C. trachomatis* have produced hybrid strains with defined regions of *C. muridarum* DNA integrated throughout the *C. trachomatis* genomes (154). These approaches have been used to map virulence factors based on differences in infectivity and immune evasion mechanisms among the two species (161).

Although the recombination machinery in the *Chlamydiaceae* family is not well characterized, *C. trachomatis* encodes genes for the RecBCD and RecFOR recombination pathways. These pathways likely function similarly to those in *Escherichia coli*, given the presence of *ruv* genes involved in Holliday junction formation and resolution (162–164). While only a few genes, such as those encoding the DNA recombination protein RecA and the exonuclease RecJ, have been functionally characterized, additional genes necessary for LGT have been identified but require further molecular characterization (136, 165, 166).

### Adaptive stress responses in *Chlamydia*

Under adverse conditions, such as those induced by immune effector molecules, nutrient deprivation, or antibiotic exposure, *Chlamydia* forms aberrant reticulate bodies (aRBs). These enlarged, non-dividing forms remain metabolically active yet non-infectious, capable of persisting long-term and potentially resuming normal development upon return to favorable conditions (7, 83, 167, 168). The formation of aRBs is considered a survival adaptation, enabling *Chlamydia* to withstand inhospitable conditions while retaining the ability to reinitiate infection. This stress response was initially observed upon exposure to β-lactam antibiotics (169). Unlike many bacteria, *Chlamydia* does not succumb to these antibiotics in a conventional bactericidal manner (111, 170, 171). Instead, aRBs form, and upon removal of the antibiotic, the non-infective aRBs transition back to EBs, maintaining viability in a “persistent” state that can last for months (171–173).

IFNγ also suppresses *Chlamydia* growth by inducing aRB formation (174). This effect is partly mediated by IFN-induced expression of host indoleamine 2,3-dioxygenase, which degrades tryptophan, critical for *Chlamydia* because, as a tryptophan auxotroph, it relies on host-derived tryptophan for survival and replication (158, 175). This amino acid scarcity triggers stress responses that arrest normal *C. trachomatis* development (176). Notably, urogenital *C. trachomatis* genovars have a partial tryptophan synthesis operon (*trpAB*), enabling them to synthesize tryptophan from indole, thereby reducing their susceptibility to IFNγ (177, 178). In addition, IFNγ activation of STAT1 leads to downregulating c-Myc (179), a transcription factor that regulates many host cell processes, including the TCA cycle and pyrimidine/purine nucleoside biosynthesis (180, 181). This nutrient limitation is another mechanism by which IFNγ promotes *C. trachomatis* persistence (182). Beyond nutritional immunity, IFNγ can restrict *Chlamydia* growth through direct pathogen-killing mechanisms. For instance, in murine cells, IFNγ limits *C. trachomatis* growth and replication with murine-specific immunity-related GTPases Iigp1/Irga6 and Irgp10 (183–186). Conversely, *C. muridarum* can circumvent these immune mechanisms in mice, underscoring the species’ adaptation to different hosts and tissues (Table 1).

Although different stressors can lead to aRB formation, the mechanisms driving persistence can differ. For instance, peptidoglycan is continuously synthesized and degraded during bacterial replication, releasing peptidoglycan-derived muropeptides. Muropeptides are highly immunogenic and recognized by the host, initiating anti-bacterial clearance pathways (187). Tryptophan depletion leads to a defect in peptidoglycan biosynthesis, which prevents muropeptide immune stimulation (188). Both mechanisms ultimately block *Chlamydia* replication and induce aRB formation, highlighting the complex role of peptidoglycan regulation in the stress response and persistence of *C. trachomatis*.

*Chlamydia* persistence represents an adaptive response to varying environmental stressors and is often linked to chronic, asymptomatic infections that may lead to severe complications in reproductive health (189). This persistence allows *Chlamydia* to evade immune surveillance and resist antimicrobial therapy, complicating treatments. The transition to and from persistence involves a complex network of regulatory pathways that respond to many cues, including host immune responses, nutrient availability, and antibiotic pressure.

## THE LIMITS OF *C. TRACHOMATIS* L2 AS A MODEL FOR *CHLAMYDIA* DISEASES

**Fig 2:**
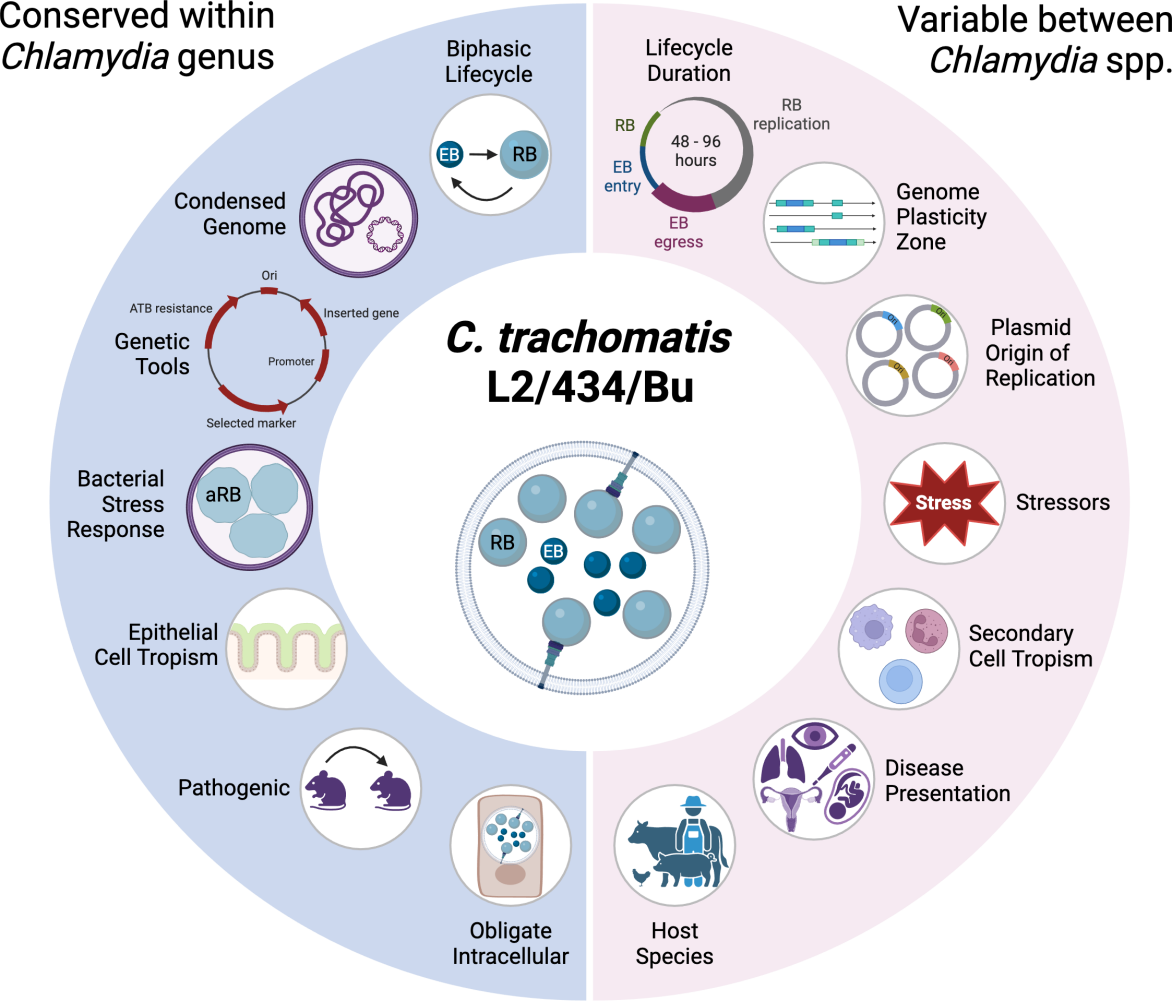
Limitations of *C. trachomatis* L2 as a model for chlamydial diseases. Traits conserved within the *Chlamydia* genus (left side) highlight why *C. trachomatis* L2/434/Bu is an effective model for studying chlamydial biology, including its unique biphasic obligate intracellular lifecycle, similarities between genomes, and preferential tropism to mucosal epithelia. In contrast, there are limits to this model based on distinctive characteristics that each *Chlamydia* species and strain possesses (right side), encompassing regions of genome variability and host and tissue tropisms. Created in https://BioRender.com.

While *C. trachomatis* L2 has been used as a prototypical *Chlamydia*, the experimental system has limitations. L2 exhibits tissue tropism that is not representative of the more clinically prevalent *C. trachomatis* urogenital and ocular genovars. These variations are primarily attributed to differences in virulence genes among *Chlamydia* genovars, which influence pathogenic mechanisms and disease outcomes. Current animal models of infection fail to accurately recapitulate *C. trachomatis* infection in humans, which limits advances in understanding and treating this pathogen (60, 190). This section will address the limits of *C. trachomatis* L2 as a model pathogen (Fig. 2).

### Genomic determinants of host and tissue tropism

*Chlamydia* species exhibit significant differences in host range. For instance, *C. psittaci* is a zoonotic pathogen that infects various host species, including birds and mammals, and can cause systemic infections in humans, known as psittacosis (191). In contrast, *C. trachomatis* is highly host-specific, primarily infecting humans and causing diseases ranging from sexually transmitted infections to trachoma (27). The ability of *Chlamydia* to infect specific hosts is influenced by complex genetic factors, including genomic variations, most notably the PZ, surface antigens, and Inc proteins. These genetic elements are crucial for mediating interactions with the host immune system and cellular machinery, thus dictating host specificity and influencing infection efficiency and immune evasion strategies (192, 193).

More than 81% of *Chlamydia* genes are either universally conserved (698 genes) or partially conserved (967 genes) across species (33, 39, 194). Among *C. trachomatis* genovars, 93% (840 genes) are universally shared, with only 2% (20 genes) unique to a single genome, emphasizing a core set of functional capabilities among these pathogens. However, the remaining 19% of genes are restricted to specific species and primarily encode functions related to metabolic processes, intracellular trafficking, and T3SS effector proteins (33). The variation in these genes likely plays a significant role in the ability of different *Chlamydia* species and serovars to infect specific cell types or tissues and adapt to different intracellular environments.

The PZ is another hotspot for genomic diversity among species (Table 1 [42, 195]). *C. trachomatis* and *C. muridarum* feature some of the largest PZs (196). The former harbors 33 open reading frames, encompassing genes responsible for biotin modification, perforin, phospholipase D (PLD)-like activity, cytotoxins, purine interconversion, and tryptophan synthesis (36, 195, 197). Notably, cytotoxin genes in *C. muridarum*, *C. psittaci*, and some *C. trachomatis* serovars are absent in *C. trachomatis* L2 (198).

*Chlamydia* species exhibit unique metabolic deficiencies, particularly in synthesizing amino acids, nucleotides, and cofactors (39). Some species-dependent metabolic differences center around genes in the PZ. The biotin synthesis pathway (bioF_2ADB) is absent in *C. trachomatis* but present in human and zoonotic pathogens like *C. pneumoniae* and *C. psittaci*, indicating that *C. trachomatis* must scavenge biotin from the host (35, 158, 199). Conversely, *C. trachomatis* genovars encode tryptophan biosynthesis genes, *trpA*, *trpB*, and *trpR*, which are absent from *C. psittaci* and *C. pneumoniae*, which impacts their response to nutritional immunity (Table 1 [55, 177, 184, 200]). This dependence on specific metabolites may contribute to the specificity of tissue and host tropism, as different *Chlamydia* species may have evolved mechanisms to exploit nutrient sources that vary among tissues.

### Animal models to study human *Chlamydia* infections

Mice and non-human primates have been used to study *Chlamydia* infections (Table 1), although each model presents unique challenges. Mice are commonly used in genital tract infections due to their ease of handling, genetic tractability, and the availability of numerous immunological tools (60). While the mouse’s upper genital tract can be infected with ocular, urogenital, and LGV *C. trachomatis* genovars, mice are not natural hosts, and the infection is rapidly cleared and does not accurately reflect the chronic natural course of human diseases (201–204). This partly stems from different immune clearance strategies between mice and humans and the limited host tropism of *C. trachomatis*. The mouse-restricted *C. muridarum* is often used as a surrogate to model acute *C. trachomatis* infections, as it mimics the natural progression from the cervical epithelium to the upper genital tract and leads to pathologies, such as hydrosalpinx, fibrosis, and infertility (205–208). However, this model does not fully replicate the human immune responses to *C. trachomatis*, particularly IFNγ responses. IFNγ controls the ascension of *C. trachomatis* to the female upper genital tract, but *C. muridarum* is less susceptible to these responses in mice (178, 209).

Non-human primates were the first animal model used to characterize *C. trachomatis* infections, providing foundational insights into chlamydial biology and pathogenesis (210). They closely represent the disease progression and immune responses observed in humans, making them a more accurate model for studying ocular and genital *Chlamydia* infections (211–213). These characteristics make non-human primates particularly valuable for preclinical testing of vaccines and therapeutics. Despite their benefits, their use is limited by high costs, fewer immunological and genetic tools, and ethical considerations (209, 214–216). However, non-human primate models have been instrumental in understanding adaptive immune responses, especially for vaccine development, providing essential insights that could not have been achieved with mouse models.

Various non-human primate animal models of *C. trachomatis* infections have been described, including baboons, marmosets, grivets, and macaques. Rhesus macaques (*Macaca mulatta*), cynomolgus macaques (*Macaca fascicularis*), and pig-tailed macaques (*Macaca nemestrina*) are most frequently used due to their anatomical and immunological similarities to humans (61, 216). Specifically, rhesus and pig-tailed macaques are often chosen for urogenital infection studies because of their anatomical similarities to the human female genital tract. For ocular trachoma research, cynomolgus macaques provide a model that closely resembles human eye infections. Additionally, cynomolgus macaques are valuable for vaccine efficacy studies because they exhibit strong humoral and cell-mediated immune responses (217). The choice of species depends on specific research objectives, with each model offering distinct advantages in immune fidelity and practical handling, enhancing their utility in studying *Chlamydia* pathogenesis and vaccine responses.

## CONCLUSIONS

The strain *C. trachomatis* L2 has provided critical insights into the unique biphasic lifecycle of *Chlamydia*, elucidating how it transitions between replicative and infectious forms (5, 6), mechanisms of DNA exchange through lateral gene transfer, and the compact genome structure of *Chlamydia* species (163). The strain has also been pivotal in identifying bacterial strategies that enable *Chlamydia* to thrive as an obligate intracellular pathogen, including manipulation of the cytoskeleton and membrane trafficking, evasion of host-induced stress responses, and maintenance of persistence (7). The genetic and molecular biology toolkits developed for *C. trachomatis* L2 have enabled the generation of similar tools for other *Chlamydia* species and provided models for testing in more clinically relevant strains, particularly genitourinary genovars, underscoring this strain’s strength as a model system (4).
